# The Form and Contagiousness of Yellow Fever

**Published:** 1879-01

**Authors:** 


The Form and Contagiousness of Yellow Fever.—Mr. Robert Lawson, Inspector General of Hospitals, after a detailed consideration of the relation of this disease to other fevers, and especially of a number of cases which occurred on board H. M. S. Bristol, sums up his conclusions as follows:

First. Yellow fever is not a disease always presenting the continued form, but is met with frequently as a remittent, and even as an opening intermittent.

Second. The sporadic cases presenting yellowness of surface and black vomit are also found to have the train of urinary symptoms characterizing yellow fever, and are consequently identical with those met with during an epidemic.

Third. In very many instances where persons in the vicinity of yellow fever cases are attacked with the disease, the facts do not admit of the exclusion of local causes, and such instances, therefore, cannot enable us to decide whether these causes or personal contagion have originated the disease; but from time to time other instances occur in which the exclusion of local causes can be assured, and in these, however extensive the exposure of susceptible individuals to the emanations from the sick may have been, the uniform result is that no communication of disease has taken place—*Lancet.*

